# Patient-, endoscopist-, and procedure-related factors associated with adenoma detection in first screening colonoscopies at a Lebanese tertiary hospital: a retrospective cohort study

**DOI:** 10.1186/s12876-026-05158-w

**Published:** 2026-07-22

**Authors:** Mariane Ibrahim Ghantous, Nadeen Zayour, Nadia Al Sabagh, Nada Mchawrab, Ogarite Kattan, Omar Eljammal, Sally El Hajj Ahmad, Maha Hoteit, Zahra Sadek, Bilal Hoteit, Nikolaos Tzenios, Mahmoud Hallal

**Affiliations:** 1 Gastroenterology Department, Faculty of Medical Sciences, Lebanese University, P.O. Box 14-6573, Beirut, Lebanon; 2 Organized Research Unit, Al Zahraa Hospital University Medical Center (ZHUMC), P.O. Box 90-361, Beirut, Lebanon; 3 PHENOL Research Program, Faculty of Public Health, Lebanese University, Section 1, P.O. Box 6573, Beirut, Lebanon; 4 INSPECT-LB (Institut National de Santé Publique, d’Épidémiologie Clinique et de Toxicologie-Liban), Beirut, 1100 Lebanon; 5 Physiotherapy Department, Faculty of Public Health, Lebanese University, P.O. Box 6573-1, Beirut, Lebanon; 6 Physical Therapy Department, Faculty of Public Health, Islamic University of Lebanon, P.O. Box 30014, Khaldeh, Lebanon; 7 Badaro Endoscopic Center, Moarbes Hospital, P.O. Box 50-223, Beirut, Lebanon; 8 Faculty of Public Health, Charisma University, London, EC1V 7QE UK; 9 Gastroenterology and Hepatology Department, Al Zahraa Hospital University Medical Center (ZHUMC), P.O. Box 90-361, Beirut, Lebanon

**Keywords:** Colorectal cancer, Colorectal cancer prevention, Colonoscopy, Adenomatous polyps, Adenoma detection rate, Polyp detection rate, Endoscopist, Performance quality

## Abstract

**Background:**

Colorectal cancer (CRC) is a leading cause of cancer-related deaths worldwide, and is largely preventable through colonoscopy, during which adenomatous polyps can be detected and removed. The adenoma detection rate (ADR) and polyp detection rate (PDR) are key quality indicators of colonoscopy quality and are inversely associated with post-colonoscopy CRC. These indicators may be influenced by patient-, physician-, and procedure-related factors. This study aimed to evaluate the ADR and PDR, identify factors influencing these indicators, and compare them among gastroenterologists at a Lebanese medical center.

**Methods:**

This observational, single-center, retrospective cohort study included 257 patients aged 50–75 years who underwent first-time screening colonoscopy over 3 years. Data on patient characteristics and colonoscopy findings were collected. ADR and PDR were calculated according to established guidelines, with ADR additionally estimated using the adenoma-to-polyp detection rate quotient (APDRQ). Descriptive statistics were applied, and chi-square or Fisher’s exact tests were used to assess associations between patient-, physician-, and procedural related factors and adenoma detection.

**Results:**

Out of 257 patients, 159 had polyps detected during colonoscopy. The overall ADR was 44.4%, and the PDR was 61.8% over the three-year study period. The ADR was 52.1% in men and 38.0% in women, whereas the PDR was 68.6% in men and 56.3% in women. Although the overall ADR and PDR met or exceeded the recommended benchmarks, disparities were noted in detection rates between males and females. Bowel preparation quality and guarantor type significantly influenced adenoma detection, whereas other factors related to patients and endoscopists had no significant effect. The estimated ADRs derived from the PDRs for five endoscopists were also high, surpassing the 25% benchmark; however, their annual estimated ADRs were relatively below this threshold.

**Conclusion:**

This study provides valuable regional data and underscores the need for standardized quality monitoring and further multicentric prospective research to optimize CRC prevention in Lebanon.

**Supplementary Information:**

The online version contains supplementary material available at https://doi.org/10.1186/s12876-026-05158-w.

## Introduction

Colorectal cancer (CRC) is a common malignancy, ranking as the third most diagnosed cancer in men and the second most common in women worldwide. It is the second leading cause of cancer related death in both males and females [1]. CRC is diagnosed most frequently between the ages of 65 and 74 years, with a surprisingly increased incidence among the youngest population starting at the age of 40 years [2]. In Lebanon, it is the fourth most reported cancer in men and the second most common in women [3], with an average age at diagnosis of 65 years for women and 66 years for men [4]. CRC progresses through the adenoma-to-carcinoma sequence, starting from normal epithelium to adenoma defined as a premalignant polypoid lesion, and eventually progresses into a malignant lesion, such as an invasive carcinoma that can metastasize [5]. Early adenomatous polyp detection and treatment are important for preventing their progression into CRC [4]. This was demonstrated by a 58% reduction in CRC mortality after screening strategies for CRC were applied [4, 6]. CRC screening aims to detect and remove early-stage cancer and precancerous lesions, such as advanced adenomas and serrated lesions [7]. The available CRC screening methods include high-sensitivity guaiac fecal occult blood tests (gFOBTs), fecal immunochemical tests (FITs), stool DNA tests, computed tomography (CT) colonography, and flexible sigmoidoscopy [2]; however, by international guidelines, colonoscopy remains the method of choice for detection and removal of all kinds of lesions, even small and asymptomatic lesions with low procedure related complication profiles [6]. In addition, colonoscopy is a cornerstone procedure in the diagnosis and treatment of various diseases, including not only CRC but also other large bowel conditions such as diverticular diseases, hemorrhoids, and inflammatory bowel diseases [8]. For CRC screening, colonoscopy is recommended every 10 years, starting at the age of 50 and continuing until the age of 75 [9].

The literature has shown that the effectiveness of CRC screening through colonoscopy heavily relies on the quality of colonoscopy procedures, which is influenced by the physician’s performance, an adequate colon examination and a proper procedural indication [6, 10]. Like other procedures, colonoscopy outcomes and performance vary according to physician skill, equipment, experience and other factors [11]. The key quality indicators include the adenoma detection rate (ADR), polyp detection rate (PDR), adenoma miss rate (AMR), cecum intubation rate (CIR), bowel preparation quality and withdrawal time [6]. These indicators are critical because, to minimize the risk of interval CRC, which is defined by CRC diagnosed after a negative screening examination and before the next recommended exam, a mean withdrawal time of more than 6 min, a CIR of 90%, and an ADR greater than 25% are needed [12].

The ADR is the primary colonoscopy quality indicator and a predictor of interval cancer, defined as the percentage of patients aged more than 50 years who are undergoing first-time screening colonoscopy with at least one pathologically identified adenoma [13, 14]. The PDR, a surrogate for the ADR due to its strong correlation, measures the proportion of patients aged ≥ 50 years with one polyp or more detected during screening colonoscopy, requiring histopathological confirmation [15]. As a colonoscopy quality indicator, the ADR is influenced by various factors, including patient demographics, bowel preparation quality, adequacy of bowel examination, and physician expertise [16]. The ADR has also been shown to be affected by patient age, as the number of adenomas increases in elderly individuals [6], and by lifestyle habits such as smoking [17]. Adequate bowel preparation improves the ADR by allowing better colonic visualization [17]. Furthermore, a proper bowel examination, defined as reaching the proximal sides of the haustral folds, rinsing mucosal surfaces, achieving adequate distention, and enhancing imaging with a withdrawal time of 6–10 min, also has a positive impact on the ADR [10]. These patient- and procedure-related characteristics contribute to ADR variability.

Research has also demonstrated that physician expertise, influenced by years of practice, the total number of colonoscopies performed, and knowledge of the endoscopic appearance of precancerous colorectal lesions, impacts the ADR, with higher rates observed among more experienced gastroenterologists [15, 17]. Therefore, a physician’s judgment and perceptions play critical roles in determining the outcomes of a colonoscopy procedure. Additionally, personality traits such as thoroughness and compulsiveness have been linked to higher ADR rates [18].

Furthermore, the ADR is considered the most critical quality measure, as a higher ADR is linked to reduced CRC incidence and mortality. Specifically, a 1% increase in the ADR lower the incidence of CRC by 3% and the mortality rate by 5% [19]. Conversely, patients screened by physicians with low ADRs face a greater risk of developing CRC [18]. Hence, the recommended adenoma detection rates are 20% for women, 30% for men, and 25% overall [14]. As the ADR and, conventionally, PDR are crucial quality indicators of colonoscopy, these parameters have been extensively studied worldwide. However, to the best of our knowledge, there is limited research on ADR and PDR in the Middle East, particularly in Lebanon. Accordingly, this study aimed to: (1) evaluate the rates of colonoscopy quality indicators, particularly the PDR and ADR; (2) examine factors influencing adenoma detection, such as body mass index (BMI), gender, smoking and alcohol intake, type of sedation, bowel preparation quality, number and type of observers, and endoscopist experience; and (3) compare the PDR and ADR among gastroenterologists in a Lebanese medical center.

## Materials and methods

### Study design

This was an observational, single-center, retrospective cohort study conducted using medical records at the Al Zahraa Hospital University Medical Center (ZHUMC), which is located in Beirut, Lebanon, between 27 March 2024 and 17 November 2024.

### Sampling and data collection procedures

Endoscopy logbooks were retrospectively reviewed to identify patients who underwent colonoscopy procedures at the endoscopy unit at ZHUMC between 1 January 2021 and 31 December 2023 and had a biopsy taken during the endoscopic procedure. The endoscopy admission sheet and colonoscopy report were subsequently examined to collect the following data: age, gender, medical and surgical history, smoking status, alcohol consumption, body mass index, medication use, type of bowel preparation and sedation, diagnosis, polyp size, and the number and location of identified adenomas and polyps.

**Fig. 1:**
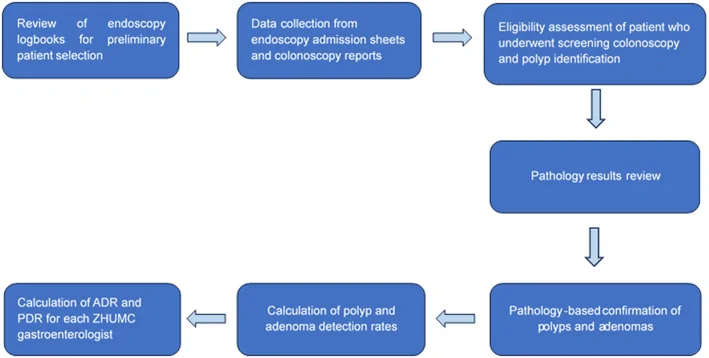
Research process of the current study

The sample population consisted of Lebanese individuals aged 50–75 years who presented as outpatients to the endoscopy unit for a first-time screening colonoscopy during the aforementioned timeframe. All colonoscopies were performed using Olympus EVIS EXERA III endoscopy systems (Olympus Medical Systems, Tokyo, Japan). Lesion detection was conducted using standard white-light imaging (WLI) alone, and image-enhanced endoscopy modalities such as narrow-band imaging (NBI) or blue light imaging (BLI) were not routinely used during examinations. The procedures were performed by board-certified gastroenterologists. The participating endoscopists had of a median of 12 years (range: 6–24 years) post-certification experience and each performed a minimum of 100 colonoscopies annually. Bowel preparation was assessed using the Boston Bowel Preparation Scale (BPPS) [20]. Patients with BBPS scores ≤ 3 (poor preparation) were excluded from the analysis due to inadequate mucosal visualization. The remaining cases were categorized as follows: 4–5 (regular), 6–7 (good), and ≥ 8 (excellent) bowel preparation. The additional exclusion criteria included a personal or family history of CRC, colorectal polyps, or adenomas, as well as a history of colonic surgery. Additional exclusions were active inflammatory bowel disease, a positive FOBT, a positive FIT, or elevated fecal calprotectin levels. Patients with incomplete colonoscopies, defined as failure to achieve cecal intubation, were also excluded. Furthermore, reports were excluded if polyps or adenomas were identified alongside other conditions, such as an anastomotic mass, Crohn’s disease, Crohn’s ileitis, or ulcerative colitis. Finally, reports documenting polyps or adenomas identified in the terminal ileum were also excluded. Figure 1 illustrates the process used in our study, outlining the steps followed to determine the desired sample size to ultimately calculate the ADR and PDR (Fig. 1).

**Fig. 2:**
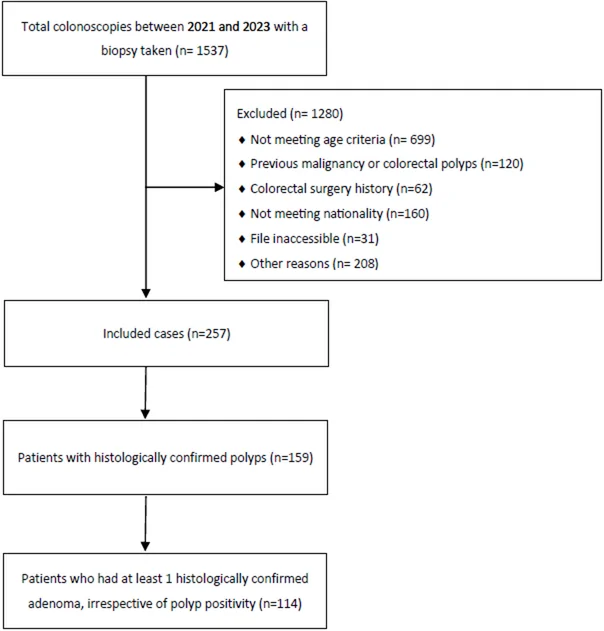
Screening and selection workflow

Among the 1,537 colonoscopy reports from colonoscopy procedures performed between January 2021 and December 2023, 257 patients met the previously established eligibility criteria, irrespective of whether a polyp or adenoma was detected. Pathology reports were reviewed to identify eligible patients with histologically confirmed polyps or adenomas. Additionally, the types, characteristics, and classifications of the biopsied polyps and adenomas were assessed and documented, with a particular focus on malignancy and dysplasia. To minimize selection bias, strict inclusion and exclusion criteria were applied. Information bias was addressed through the use of standardized data collection forms and thorough training of the study team, which was supervised by the research assistant (NZ). Ultimately, a total of 159 patients were identified as eligible, each having at least one histologically confirmed polyp or adenoma (Fig. 2).

### Ethical approval

The study received approval from the Institutional Review Board at ZHUMC (reference number: 9/2024) and followed the ethical guidelines of the Declaration of Helsinki. Prior to retrospective data collection, written informed consent was obtained from the endoscopists to allow access to their files. All patients provided informed consent permitting the use of their colonoscopy data, with anonymity maintained. Data access was restricted solely to the research assistant (N.Z.) to ensure confidentiality and prevent unauthorized use. After data collection ended, the study team did not have any access to any information that could reveal the identities of individual patients.

### Statistical analysis

The ADR and PDR were calculated according to established recommendations [13–15]. The ADR was also estimated indirectly through the PDR through calculating the adenoma-to-polyp detection rate quotient (APDRQ) for each endoscopist, which is the ratio of the ADR to the PDR. The average APDRQ across all endoscopists was then used as a conversion factor to estimate individual ADRs [17]. This method was employed to provide ADR estimates for endoscopists with limited histological data. Finally, the annual ADR for each physician was also calculated.

To analyze the data, descriptive statistics were used to summarize demographic, patient-related, and physician-related variables. The chi-square test or Fisher’s exact test was used to compare categorical variables. Chi-square analysis assessed the association between patient and physician risk factors and adenoma detection. A p-value of less than 0.05 was considered statistically significant. All analyses were conducted via SPSS version 22.

## Results

### Characteristics of the study population

#### Sociodemographic characteristics

The patient population in this study had a mean age of 62.3 years, with an equal distribution of males (49.7%) and females (50.3%). The majority were married (90.6%), and only a small percentage were single (7.5%) or divorced (1.9%). With respect to bowel movement issues, most patients reported no abnormalities (54.1%), whereas others reported constipation (23.9%), diarrhea (17.0%), or a combination of constipation and diarrhea (5.0%). The anthropometric data revealed a median weight of 75 kg and a median height of 168 cm. The median BMI was 26.0 kg/m², suggesting a moderate level of overweight across the groups. In terms of lifestyle factors, the majority of the study population were smokers (61.0%), while only 5% reported alcohol consumption. A significant portion of the patients (73.6%) were on some form of medication, with the largest group (40.3%) taking 1 to 2 medications. Regarding medical and procedural histories, most patients (79.2%) had 1 to 3 diseases, with a smaller group (16.4%) suffering from more than 3 conditions. A substantial portion (79.2%) had been previously hospitalized, and the majority (65.4%) had undergone 1 to 2 procedures (Table 1).

**Table 1:** Demographic characteristics of the study population

Characteristic		*n* (%)
Age	62.3 (56.0, 68.0)	
Gender	Male	79 (49.7%)
	Female	80 (50.3%)
Marital status	Single	12 (7.5%)
	Married	144 (90.6%)
	Divorced	3 (1.9%)
Bowel movement	No abnormality	86 (54.1%)
	Constipation	38 (23.9%)
	Diarrhea	27 (17.0%)
	Constipation and diarrhea	8 (5.0%)
Weight (kg)	75 (65, 83)	
Height (cm)	168 (160, 174)	
BMI (kg/m^2^)	26.0 (23.4, 29.1)	
Smoking	Yes	97 (61.0%)
	No	62 (39.0%)
Alcohol	Yes	8 (5.0%)
	No	151 (95.0%)
Medication intake	Yes	117 (73.6%)
	No	42 (26.4%)
Medication intake (as categories)	None	42 (26.4%)
	1 to 2 medications	64 (40.3%)
	3 or more medications	53 (33.3%)
Medical history	None	7 (4.4%)
	1 to 3 diseases	126 (79.2%)
	More than 3 diseases	26 (16.4%)
Previous hospitalization	Yes	126 (79.2%)
	No	33 (20.8%)
Procedural history	None	33 (20.8%)
	1 to 2 procedures	104 (65.4%)
	3 or more procedures	22 (13.8%)

*BMI* Body Mass Index

#### Medical and health characteristics of the study population

A variety of conditions were identified, with hypertension being the most prevalent, affecting 47.2% of individuals, whereas diabetes affected 30.8% of the patients. Other conditions observed in the group included heart disease (25.8%), thyroid conditions (6.9%), and dyslipidemia (6.9%). A smaller proportion of patients reported cancers other than CRC (6.3%), neurological diseases (3.1%), kidney disease (2.5%), and lung disease (2.5%). Blood disorders and bone diseases were rare, each affecting only 1.3% of patients. The majority of the patient population did not report these conditions, indicating that while some chronic health conditions were common, others were less prevalent. Patients may have more than one condition; thus, percentages do not sum to 100%.

#### Procedural history of the study population

The procedures performed during previous hospitalizations varied across the patient population. The most common procedures were cardiac interventions (14.5%), orthopedic surgeries (13.8%), gynecological procedures (13.2%), and hernia repairs (13.2%). Other notable procedures were cholecystectomy (10.1%), gastroscopy (8.2%), and procedures performed for unknown or unclear reasons (7.5%). Less frequent procedures included appendectomy (6.3%), urological procedures (5.0%), hemorrhoidectomy (2.5%), and cancer-related surgeries such as mastectomy (1.9%) and tumor resection (1.3%). Many patients did not undergo any of these procedures, with a substantial portion having no prior procedural history (Table S1).

### Aspects of colonoscopy procedures performed in the study population

#### Colonoscopy procedural characteristics

The procedures performed primarily included colonoscopy and gastroscopy. A majority of patients (70.4%) underwent both procedures, while 29.6% had only a colonoscopy. Most of the procedures were conducted in 2023 (41.5%), followed by 2022 (35.2%) and 2021 (23.3%). The majority of these procedures (94.3%) were performed by three observers, the endoscopist, the gastrointestinal (GI) fellow, and a technician, whereas a smaller proportion of the colonoscopies (5.7%) involved only two observers, the endoscopist and the technician. The distribution of the BBPS scores, was relatively even, with 48.4% of the procedures scoring between 6 and 7, 46.5% scoring 8 or above, and 5% scoring between 4 and 5. The reparation quality was reported to be good in 48.4% of cases and excellent in 46.5%, with only a small fraction (5%) rated as regular (Table 2).

**Table 2:** Colonoscopy procedural characteristics

Characteristics		*n* (%)
Procedure(s) performed	Colonoscopy only	47 (29.6%)
	Colonoscopy and Gastroscopy	112 (70.4%)
Year of procedure	2021	37 (23.3%)
	2022	56 (35.2%)
	2023	66 (41.5%)
Participant types	Endoscopist, fellow, and technician	150 (94.3%)
	Endoscopist and technician	9 (5.7%)
Number of overall observers	2 observers	9 (5.7%)
	3 observers	150 (94.3%)
BBPS Score	4 to 5	8 (5.0%)
	6 to 7	77 (48.4%)
	8 or above	74 (46.5%)
Preparation quality	Regular	8 (5.0%)
	Good	77 (48.4%)
	Excellent	74 (46.5%)

*BBPS* Boston Bowel Preparation Scale

#### Colonoscopy procedure coverage of the study population

The majority of patients (77.4%) paid for the colonoscopy procedure out of pocket. Other sources of coverage included medical insurance (10.7%), hospital employees (5.7%), and military and other security forces (4.4%). A small proportion were supported through donations (1.3%), whereas coverage by municipalities and unions accounted for 0.6% of the cases.

#### Colonoscopy findings for the study population

**Fig. 3:**
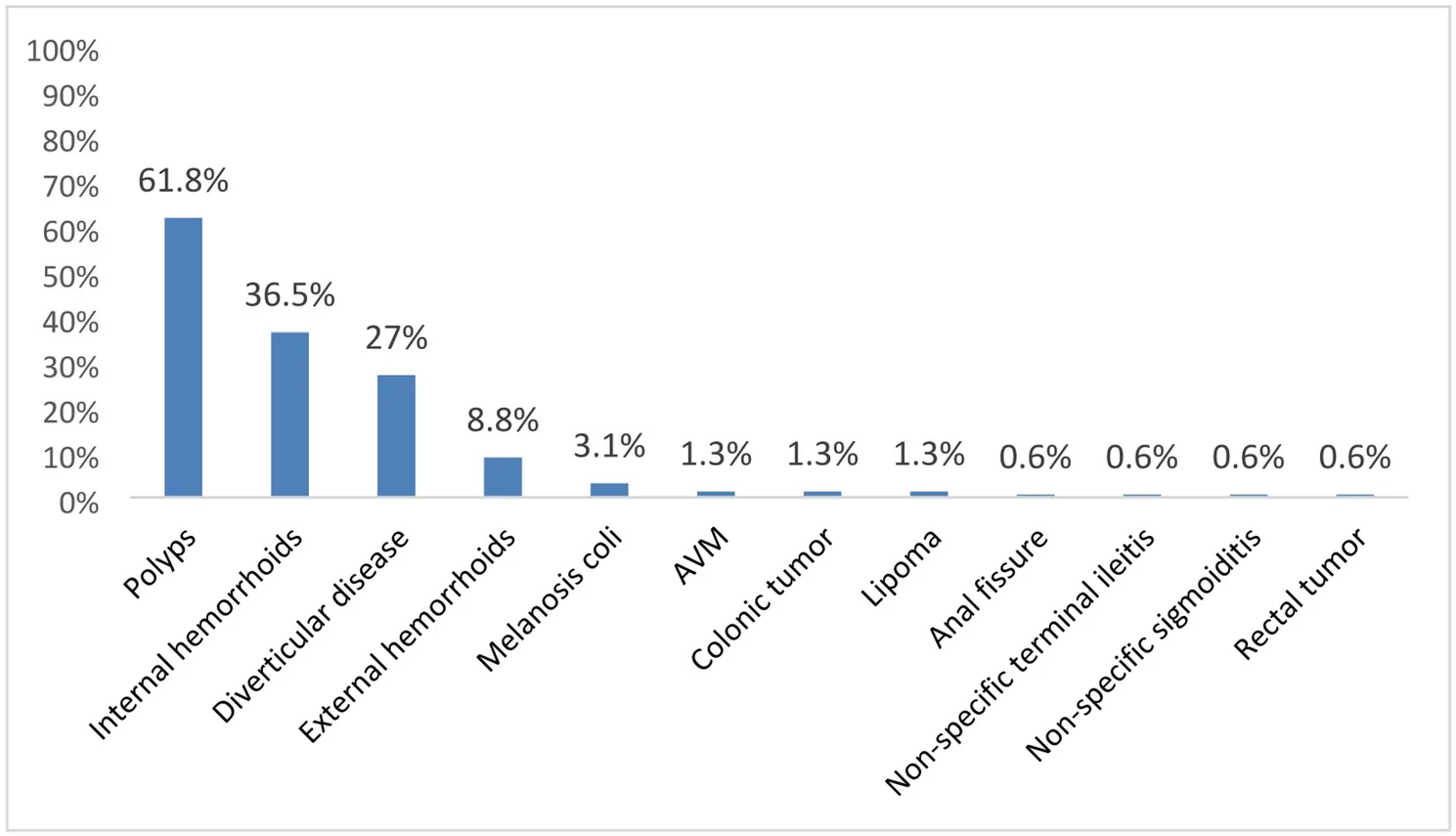
Bar chart showing the percentage of colonoscopic findings among the study population. Multiple findings were possible per patient; therefore, percentages do not total 100%. AVM: arteriovenous malformation

Colonoscopy revealed a range of conditions, with polyps identified in 61.8% of the cases. Other notable findings included: internal hemorrhoids (36.5%), diverticular disease (27%), and external hemorrhoids (8.8%). A smaller proportion of patients had melanosis coli (3.1%), colonic tumors (1.3%), lipomas (1.3%), and arteriovenous malformations (AVMs) (1.3%). Additionally, there were a few cases of anal fissure, rectal tumors, and non-specific terminal ileitis or sigmoiditis, each accounting for 0.6% of the cases. Patients may have more than one condition; thus, percentages do not sum to 100% (Fig. 3).

#### Colonoscopy description of identified polyps in the study population

The characteristics of the polyps found during colonoscopy revealed that most patients (83.6%) had between 1 and 3 polyps, while 16.4% had multiple polyps. In terms of polyp size, 79.2% were diminutive (1–5 mm), 31.4% were small (6–9 mm) and 12.6% were large (≥ 10 mm). The most common locations for polyps were the sigmoid colon (35.2%), ascending colon (32.7%), and descending colon (29.6%), with smaller proportions in regions such as the anus (5%) and anorectal area (2.5%). In terms of polyp description, 46.5% were deemed diminutive, 35.2% were sessile, and 14.5% were pedunculated (Table 3).

**Table 3:** Characteristics of identified polyps during colonoscopy

Characteristics		*n* (%)
Number of polyps	1–3 polyps	133 (83.6%)
	> 3 polyps (multiple)	26 (16.4%)
Size*	Polyp size of 1–5 mm (diminutive)	126 (79.2%)
	Polyp size of 6–9 mm (small)	50 (31.4%)
	Polyp size of ≥ 10 mm (large)	20 (12.6%)
Location*	Sigmoid	56 (35.2%)
	Ascending colon	52 (32.7%)
	Descending colon	47 (29.6%)
	Rectum	39 (24.5%)
	Transverse colon	31 (19.5%)
	Cecum	23 (14.5%)
	Anus	8 (5.0%)
	Anorectal	4 (2.5%)
Colonoscopy report polyp description*	Diminutive	74 (46.5%)
	Sessile	56 (35.2%)
	Pedunculated	23 (14.5%)
	Subpedunculated	9 (5.7%)
	Flat	4 (2.5%)
	Neoplastic	3 (1.9%)
	Serrated	1 (0.6%)

* Patients may have polyps of multiple sizes, different locations, and descriptions; thus, percentages do not sum to 100%

#### Management of identified polyps during colonoscopy

Following polyp identification during colonoscopy, management strategies largely included biopsy and polypectomy, with both performed in 98.7% of cases. The predominant resection technique was cold forceps (71.7%), followed by cold snare (20.8%), hot snare (18.2%), and endoscopic mucosal resection (EMR) (4.4%). Only a small percentage of patients (1.3%) underwent biopsy alone, without polypectomy resection. In terms of biopsy source, most specimens were obtained directly from polyps (71.1%), while others were taken from different parts of the colon, including the sigmoid colon (5.7%), ascending colon (4.4%), cecum (2.5%), and descending colon (2.5%). The resection technique and source of biopsy may be more than one; thus, percentages do not sum to 100%.

### Histopathological confirmation of identified polyps

Biopsy pathology revealed that 71.7% of the polyps were adenomas, 30.2% were other types, and interestingly only 1.9% were adenocarcinomas. Pathology reports described the majority (71.7%) as adenomatous, with 30.2% labeled as hyperplastic, 9.4% as inflammatory, and a smaller number as tubular, tubulovillous, or ulcerated. Patients may have more than one pathology result and description; thus, percentages do not sum to 100%. With respect to the grade of dysplasia, most polyps (69.8%) had low-grade dysplasia, with only a small number exhibiting moderate or high-grade dysplasia (0.6% and 1.3%, respectively), and 28.9% showed no dysplasia.

### Adenoma and polyp detection rates

#### Adenoma and polyp detection rates of the study population

The overall ADR for the study was 44.4%, with 114 out of 257 patients having adenomas detected. The overall PDR was 61.8%, with 159 out of 257 patients having polyps detected. When broken down by gender, the ADR for males was 52.1% (60 out of 115), while for females, it was 38.0% (54 out of 142). The PDR for males was 68.6% (79 out of 115), while for females, it was 56.3% (80 out of 142). Looking at trends over the years, the ADR decreased slightly from 49.2% in 2021 to 42.5% in 2023, with 29/59, 37/85, and 48/113 adenomas detected in those respective years. The PDR trend also showed a decrease, from 62.7% in 2021 to 58.4% in 2023, with 37/59, 56/85, and 66/113 polyps detected in those years (Table 4).

**Table 4:** ADR and PDR of the study population

	*n*/*n*	%
Adenoma Detection Rate (overall)	114/257	44.4%
Polyp Detection Rate (overall)	159/257	61.8%
ADR among		
Males	60/115	52.1%
Females	54/142	38.0%
PDR among		
Males	79/115	68.6%
Females	80/142	56.3%
ADR Trend		
2021	29/59	49.2%
2022	37/85	43.5%
2023	48/113	42.5%
PDR Trend		
2021	37/59	62.7%
2022	56/85	65.8%
2023	66/113	58.4%

#### Estimated adenoma and polyp detection rates of ZHUMC endoscopists

The actual ADRs of the five ZHUMC endoscopists varied significantly. Endo50 achieved the highest actual ADR at 58%, indicating exceptional performance. Endo1 followed by a strong ADR of 47%, suggesting effective detection capabilities. In contrast, Endo42 and Endo60 had lower ADRs of 41% and 39%, respectively. Endo64 also recorded an ADR of 39%, aligning with Endo60 at the lower end of the performance spectrum. The PDR showed a similar distribution pattern. Endo1 and Endo 50 achieved the highest PDR at 73%, followed by Endo64 at 61%, and Endo60 at 60%. In contrast, Endo42 had a notably lower PDR of 58%. The APDRQ, calculated as the ADR/PDR ratio, indicates the relative efficiency of adenoma detection compared to polyp detection. Endo50 exhibited a remarkably high APDRQ of 79.4%, highlighting a significantly greater rate of adenoma detection relative to polyp detection. Endo42 also showed strong performance with an APDRQ of 70.6%. Endo60 had a percentage of 65%, followed by Endo1 at 64.3%, and Endo64 at 63.9%. These values suggest that, for most of the gastroenterologists assessed, adenoma detection rates substantially exceed polyp detection rates. The estimated ADR further supported these findings, with values calculated using a conversion factor of 0.718. In this case, Endo1 and Endo 50 maintained the highest estimated ADR at 52.4%, followed by Endo64 at 43.7% and Endo60 at 43%. These estimated rates provide a predictive outlook on ADR performance. Annualized estimates were then calculated by dividing the estimated ADR by 3 to reflect the expected yearly performance of each gastroenterologist. Even after annualization, Endo1 and Endo50 continued to have the highest estimated ADR per year (17.4%), while Endo60 and Endo42 had lower annual ADR estimates of 14.3% and 13.8%, respectively (Table 5).

**Table 5:** Estimated ADRs for five ZHUMC endoscopists

Endoscopist Code	Patients undergoing screening procedures	Patients with at least 1 histologically confirmed adenoma	Patients with at least 1 histologically confirmed polyp	Actual ADR	PDR	APDRQ	Estimated ADR (using a conversion factor of 0.718)	Estimated ADR per year (divided by 3)
Endo1	19	9	14	47%	73%	64.3%	52.4%	17.4%
Endo42	17	7	10	41%	58%	70.6%	41.6%	13.8%
Endo50	26	15	19	58%	73%	79.4%	52.4%	17.4%
Endo60	99	39	60	39%	60%	65%	43%	14.3%
Endo64	31	12	19	39%	61%	63.9%	43.7%	14.5%

### Exploring factors affecting adenoma detection

To compare patients with and without adenomas, several factors were analyzed for potential associations. The average patient age and gender distribution were not significantly different between the adenoma-negative and adenoma-positive groups. The average BMI was slightly greater in the adenoma-positive group (26.6 kg/m^2^ ± 4.1) than in the adenoma-negative group (25.8 kg/m^2^ ± 4.1), but the difference was not statistically significant (*p* = 0.257). Similarly, smoking status, alcohol consumption, and the presence of medical conditions were not significantly associated with adenoma detection. However, bowel preparation quality was significantly linked to adenoma detection, with excellent preparation being more common in the adenoma-positive group (*p* = 0.033). Interestingly, patients who self-paid were more likely to have adenomas detected (*p* = 0.020). No significant associations were found between adenoma detection and the year of procedure, number of observers, or endoscopist-related factors including endoscopist age, years of experience, continuing medical education (CME) credits, or membership in the Lebanese Society of Gastrointestinal Endoscopy (LSGE). Hence, better bowel preparation quality and self-payment status were significantly associated with greater adenoma detection, whereas patient and endoscopist demographics, endoscopist experience, and procedural characteristics had no significant impact (Table 6).

**Table 6:** Associations between patient, procedural and physician factors, and adenoma detection

		Adenoma negative	Adenoma positive	*p*- value
Patient age		62 ± 7.1	62 ± 6.9	0.620
BMI (kg/m^2^)		25.8 ± 4.1	26.6 ± 4.1	0.257
Gender	Male	19	60	0.237
	Female	26	54	
Smoking	Yes	28	69	0.843
	No	17	45	
Alcohol	Yes	2	6	0.832
	No	43	108	
Medical history	None	2	5	0.810
	1 to 3 diseases	37	89	
	More than 3 diseases	6	20	
Preparation quality	Regular	4	4	**0.033**
	Good	27	50	
	Excellent	14	60	
Guarantor type	Self-payer	34	89	**0.020**
	Hospital employees	0	9	
	Medical insurance	5	12	
	Military and other security forces	5	2	
	Municipalities and unions	1	0	
	Donations	0	2	
Year of procedure	2021	29	8	0.423
	2022	37	19	
	2023	48	18	
Number of overall observers	2 observers	2	7	0.667
	3 observers	43	107	
Participant types	Endoscopist, fellow, and technician	43	107	0.677
	Endoscopist and technician	2	7	
Endoscopist age		52 ± 12.9	53 ± 13.2	0.778
Endoscopist CME credits	≤ 25 credits	24	70	0.351
	> 25 credits	21	44	
Endoscopist LSGE membership	Yes	45	112	0.371
	No	0	2	

Bold values indicate statistical significance (*p* < 0.05)

*BMI *Body mass index, *CME* Continuing medical education, *LSGE* Lebanese Society of Gastrointestinal Endoscopy

## Discussion

This study evaluated the ADR and PDR among outpatients at a tertiary healthcare center, with a focus on performance trends and associated influencing factors. Our study revealed an overall ADR of 44.4%, which is notably higher than the generally recommended benchmark of 25% and exceeds the gender-specific thresholds [14]. The overall PDR was 61.8%, which is consistent with the expected range for high-quality colonoscopy [15]. Interestingly, males had slightly higher ADRs and PDRs than females (52.1% vs. 38.0% and 68.6% vs. 56.3%, respectively). These gender-based differences in detection rates were not statistically significant (*p* = 0.237) but are in line with literature suggesting that men tend to have a higher prevalence of adenomas, likely due to hormonal, behavioral, and lifestyle factors [6]. Overall, these findings reflect generally good colonoscopic performance, which is a positive outcome, particularly given the well-established relationship between increased ADR and reductions in both CRC incidence and mortality [19]. These findings are consistent with those reported by Tarhini et al. [21] in a Lebanese cohort, who demonstrated a PDR of 70% and an ADR of 52% among patients undergoing colonoscopy. Another Lebanese study reported an ADR of 20.5% with age above 50 years and male sex emerging as significant predictors of adenoma detection [5]. Although direct comparisons should be interpreted with caution, as that study included both younger and older patients and was not restricted to average-risk individuals undergoing first-time screening colonoscopy, the substantially higher ADR observed in our cohort may be attributable to the application of stricter inclusion criteria, the exclusion of surveillance and diagnostic colonoscopies, and adherence to contemporary quality standards for ADR calculation. In contrast, Almadi et al. [22] reported a PDR of 20.8% and an ADR of 8.1% among 2,654 colonoscopies conducted in Saudi Arabia. These differences may be explained by variations in patient selection, screening practices, endoscopist performance, bowel preparation quality, and methodological approaches. When yearly trends were examined, adenoma detection showed a modest decline between 2021 and 2023. Importantly, the number of patients varied across years due to the impact of the COVID-19 pandemic in 2021 and 2022, which limits the validity of year‒to-year comparisons. Despite the lack of statistical significance, this evolving pattern may reflect improved awareness and identification of diminutive polyps, enhanced imaging technologies, or better bowel preparation practices. Nevertheless, these trends warrant continued monitoring through longitudinal assessment.

The pathological characteristics of the detected lesions support the effectiveness of screening in this outpatient population. Among the 159 patients with polyps, 114 (71.7%) had confirmed adenomas, the majority of which exhibited low-grade dysplasia (69.8%). Additionally, three cases of adenocarcinoma (1.9%) were identified, highlighting the potential for early cancer detection and timely intervention. These findings are consistent with the adenoma–carcinoma sequence and underscore the value of screening even among asymptomatic individuals [2, 5]. In terms of polyp characteristics, the majority were diminutive in size (1–5 mm), exhibited a sessile morphology, and were identified primarily in the sigmoid colon, ascending colon, and descending colon. Given that diminutive and sessile polyps are easily missed, these results emphasize the critical role of meticulous mucosal inspection and proper withdrawal techniques during colonoscopy [10]. In our cohort, 98.7% of polyps were managed through biopsy and polypectomy. Cold forceps were used in 71.7% of the patients, whereas cold snare resection, recommended by the European Society of Gastrointestinal Endoscopy (ESGE) [23] as the preferred method for small, sessile polyps, was employed in 20.8% of the patients. These findings indicate strong adherence to recommended polyp management practices, particularly the widespread use of cold forceps for small polyps. However, the relatively lower use of cold snare resection, despite its guideline-supported role, suggests an opportunity to further align clinical practice with evidence-based standards to optimize resection quality and completeness. Interestingly, endoscopist-related factors such as years of experience, CME credits, and membership in the LSGE showed no statistically significant impact on adenoma detection. This finding aligns with previous studies that emphasize endoscopic technique and behavior over formal qualifications [15–17]. Similarly, the number and type of observers present during procedures did not significantly influence adenoma detection, although prior research has suggested that a greater number of observers may enhance detection through collaborative viewing and educational reinforcement [15, 17, 24]. One unexpected finding was the significant association between guarantor type and lesion detection, with self-paying patients having a higher adenoma detection rate than those covered by third-party payers. This finding should be interpreted with caution, as the underlying reasons for this association could not be determined from the available data. It is possible that unmeasured differences in patient characteristics, healthcare-seeking behaviors, screening practices, health awareness, or other socioeconomic factors may have contributed to the observed association. Residual confounding cannot be excluded, and therefore a causal relationship should not be inferred. Further studies are needed to explore the mechanisms underlying this finding and to determine whether it persists after accounting for potential confounders.

One of the most impactful procedural factors in our analysis was bowel preparation quality, which was assessed using the BBPS. Patients with regular or good preparation had significantly lower ADRs than those with excellent preparation, confirming the well-established link between a clean mucosal surface and improved polyp and adenoma visualization [17]. Importantly, patients with poor bowel preparation ability were excluded from the study, which explains the high percentage (94.9%) of procedures with BBPS scores ≥ 6, indicating that optimal bowel preparation was widely achieved in this population. Our study revealed no significant associations between age, gender, smoking status, alcohol consumption, or BMI and adenoma detection. This contrasts with previous research that identified increasing age, male gender, and smoking as established adenoma risk factors, and obesity as a known risk enhancer for colorectal cancer [6, 7, 17, 25, 26].

In this study, the calculated APDRQ was 0.718, whereas the value reported by [17], was 0.4085. The estimated ADRs were derived from PDRs via the APDRQ multiplier, but only for endoscopists who had at least 15 patients who underwent screening procedures during the study period (2021–2023). Among the 26 endoscopists included in the study, only 5 met these criteria. Among these five practitioners, notable variation in ADRs was observed. This variability supports previous findings that detection rates can vary widely among physicians, often due to differences in technique, attention to detail, withdrawal quality, and individual traits such as thoroughness and meticulousness [15–17]. The actual ADRs for all five physicians were well above the recommended benchmark of 25%, representing a strong indicator of detection performance. This high performance may, in part, be attributed to the fact that the majority of procedures in the study were performed by more experienced endoscopists. Interestingly, the estimated ADRs based on the conversion factor of 0.718 revealed a different ranking for the endoscopists. Endo50, despite having the highest actual ADR, ranked the same as Endo1 in terms of the estimated ADR. This discrepancy highlights the limitations of using PDR-derived estimates to substitute for pathology-confirmed ADRs, as they may not accurately reflect true detection performance in all cases [15]. These findings reinforce the recommendation that the ADR should remain the gold standard quality indicator for colonoscopy, whereas the PDR may be used only when pathology data are unavailable [14].

A key strength of this study is that it is the first in Lebanon to rigorously adhere to international guidelines for calculating the ADR. In line with established recommendations, we limited the study population to average-risk individuals aged 50–75 years who underwent screening colonoscopy. Additionally, strict exclusion criteria were applied, such as the exclusion of patients with poor bowel preparation, diagnostic or surveillance indications, incomplete procedures, etc., to ensure accurate and meaningful ADR measurements.

This study, however, has several limitations, primarily related to its retrospective design, relatively small sample size, and single-center setting, which may restrict the generalizability of the findings. The limited sample size may have reduced the statistical power to detect certain associations, while the single-center design may reflect local patient characteristics, referral patterns, endoscopic practices, and operator expertise that may differ from those of other institutions. In particular, the relatively high ADR and PDR observed in this study may be explained by several factors. First, the study was conducted in a tertiary referral center where endoscopists may have greater experience and adherence to quality standards, potentially contributing to higher detection rates and therefore may not be representative of ADRs achieved in other clinical settings. Second, the inclusion of first-time screening colonoscopies may have selected a population with a higher prevalence of undiagnosed lesions. In addition, variability in bowel preparation quality and careful inspection techniques may have further influenced detection rates. Consequently, caution is warranted when extrapolating these findings to broader populations. Future multicenter, prospective studies with larger and more diverse populations are needed to validate these findings, explore inter-operator variability, and optimize interventions aimed at improving ADR and PDR. Nevertheless, important procedural variables known to influence adenoma detection, including withdrawal time and cecal intubation time, could not be assessed because these variables were not consistently recorded in the medical records. Both indicators are recommended by the ESGE and the American Society for Gastrointestinal Endoscopy (ASGE) as key measures of colonoscopy quality and have been shown to be independently associated with adenoma detection. Consequently, the absence of these data limited our ability to determine whether variations in ADR and PDR were attributable to differences in endoscopist performance, procedural quality, or patient-related factors. This limitation may have introduced residual confounding and should be considered when interpreting the observed detection rates. Future interventions should ensure systematic documentation of these parameters to allow a more comprehensive assessment in accordance with international guidelines. Furthermore, the use of estimated ADRs derived from PDR also presents inherent limitations, particularly given the observed discrepancy in ranking between actual and estimated ADRs among individual endoscopists. Additionally, estimated values could not be calculated for all 26 physicians in the unit due to variability in patient volume per endoscopist, an issue further influenced by the COVID-19 pandemic, which led to lower patient numbers in 2021 and 2022 than in 2023.

## Conclusions

This study demonstrates that ADR and PDR meet or exceed expected quality standards. Bowel preparation quality significantly influenced ADR, and variability in detection rates among endoscopists highlights the need for ongoing quality monitoring and feedback. These findings underscore the importance of optimizing colonoscopy quality to improve CRC screening outcomes, support further efforts to standardize practice and reduce inter-operator variability.

## Supplementary Information

Supplementary Material 1. Table S1: Patient procedural history.

## Data Availability

All data generated or analyzed during this study are included in this published article [and its supplementary information files].

## Abbreviations

CRC: Colorectal cancer
gFOBTs: Guaiac fecal occult blood tests
FITs: Fecal immunochemical tests
CT: Computed tomography
ADR: Adenoma detection rate
PDR: Polyp detection rate
AMR: Adenoma miss rate
CIR: Cecal intubation rate
BMI: Body mass index
ZHUMC: Al Zahraa Hospital University Medical Center
WLI: white-light imaging
NBI: Narrow-band imaging
BLI: Blue light imaging
BBPS: Boston Bowel Preparation Scale
APDRQ: Adenoma-to-polyp detection rate quotient
GI: Gastrointestinal
AVMs: Arteriovenous malformations
EMR: Endoscopic mucosal resection
CME: Continuing medical education
LSGE: Lebanese Society of Gastrointestinal Endoscopy
ESGE: European Society of Gastrointestinal Endoscopy
ASGE: American Society for Gastrointestinal Endoscopy
