# Supplementary material for: Patient-, endoscopist-, and procedure-related factors associated with adenoma detection in first screening colonoscopies at a Lebanese tertiary hospital: a retrospective cohort study
Source: BMC Gastroenterol. 2026 Jul 22;26:663. doi: 10.1186/s12876-026-05158-w (PMC13679282; doi:10.1186/s12876-026-05158-w)
Supplement: Supplementary file 1 [file 12876_2026_5158_MOESM1_ESM.docx]

**Table S1. Patient procedural history**

| **Procedural History*** | **n (%)** |
| --- | --- |
| Cardiac intervention | 23 (14.5%) |
| Orthopedic surgeries | 22 (13.8%) |
| Gynecological procedures | 21 (13.2%) |
| Hernia repair | 21 (13.2%) |
| Cholecystectomy | 16 (10.1%) |
| Gastroscopy | 13 (8.2%) |
| Unknown/unclear procedure | 12 (7.5%) |
| Appendectomy | 10 (6.3%) |
| Urological procedure | 8 (5.0%) |
| Cataract surgery | 5 (3.1%) |
| Cyst or abscess removal | 5 (3.1%) |
| Prostate surgery | 5 (3.1%) |
| Thyroidectomy | 5 (3.1%) |
| Tonsillectomy | 4 (2.5%) |
| Hemorrhoidectomy | 4 (2.5%) |
| Mastectomy partial or total | 3 (1.9%) |
| Vascular surgery | 3 (1.9%) |
| Sub–mucous resection (SMR) | 3 (1.9%) |
| Tumor resection | 2 (1.3%) |
| Anal fissure repair | 2 (1.3%) |
| Lithotripsy | 2 (1.3%) |
| Dialysis | 1 (0.6%) |
| Esophageal dilation surgery | 1 (0.6%) |
| Lobectomy | 1 (0.6%) |
| Plastic surgery | 1 (0.6%) |
| Rectal prolapse | 1 (0.6%) |
| Splenectomy | 1 (0.6%) |

*Patients may have undergone more than more procedure; thus, percentages do not sum to 100%.
